# ECG-RNG: A Random Number Generator Based on ECG Signals and Suitable for Securing Wireless Sensor Networks

**DOI:** 10.3390/s18092747

**Published:** 2018-08-21

**Authors:** Carmen Camara, Pedro Peris-Lopez, Honorio Martín, Mu’awya Aldalaien

**Affiliations:** 1 Department of Computer Science, University Carlos III of Madrid, 28911 Leganés, Spain; pperis@inf.uc3m.es; 2 Department of Electronic Technology, University Carlos III of Madrid, 28911 Leganés, Spain; hmartin@ing.uc3m.es; 3 Higher Colleges of Technology, Abu Dhabi Women’s College, Abu Dhabi 41012, United Arab Emirates; maldalaien@hct.ac.ae

**Keywords:** Wireless Sensor Networks (WSNs), Electrocardiogram (ECG) sensor, Random Number Generators (RNGs), wavelet

## Abstract

Wireless Sensor Networks (WSNs) are a promising technology with applications in many areas such as environment monitoring, agriculture, the military field or health-care, to name but a few. Unfortunately, the wireless connectivity of the sensors opens doors to many security threats, and therefore, cryptographic solutions must be included on-board these devices and preferably in their design phase. In this vein, Random Number Generators (RNGs) play a critical role in security solutions such as authentication protocols or key-generation algorithms. In this article is proposed an avant-garde proposal based on the cardiac signal generator we carry with us (our heart), which can be recorded with medical or even low-cost sensors with wireless connectivity. In particular, for the extraction of random bits, a multi-level decomposition has been performed by wavelet analysis. The proposal has been tested with one of the largest and most publicly available datasets of electrocardiogram signals (202 subjects and 24 h of recording time). Regarding the assessment, the proposed True Random Number Generator (TRNG) has been tested with the most demanding batteries of statistical tests (ENT, DIEHARDERand NIST), and this has been completed with a bias, distinctiveness and performance analysis. From the analysis conducted, it can be concluded that the output stream of our proposed TRNG behaves as a random variable and is suitable for securing WSNs.

## 1. Introduction

We are in the era of the Internet of Things (IoT), where all kinds of devices and sensors are connected to the Internet. There is a wide variety of applications/sectors that can benefit from this technology, but it can turn into a nightmare if security does not play a critical role [1,2]. This is even more critical, if possible, in particular sectors like the health-care sector, where sensors are in or on a subject’s body, and a cybersecurity attack could have dramatic consequences. The reader should note that the new generations of implanted medical devices (e.g., pacemakers or insulin pumps) are already equipped with wireless connectivity and can be remotely accessed [3,4]. The security risks of these medical devices have been recently scrutinized, and the results show certain security pitfalls in some commercial devices [5].

Wireless Sensors Networks (WSNs) are one of technologies that supports the IoT paradigm. In a nutshell, a WSN consists of a large number of nodes, each one of them equipped with a sensor. The nodes sense their environment (e.g., humidity or pressure), communicate with each other and transmit the collected data to a gateway with Internet connectivity [6]. A particular case of WSNs is the Wireless Body Area Networks (WBANs) in which the nodes are in or on the human body and collect its vital signals [7]; in this case, the gateway is often implemented by a smartphone. The collected signals can be used for health applications or, as some authors have recently proposed, for security purposes [8]. The latter is the objective of this proposal and is part of the dependability aspects of WSNs and should be addressed for a widespread adoption of this technology [9].

As already mentioned, to prevent, or at least hinder, cybersecurity attacks, security mechanisms must be added at the design phase of the sensors [10]. Regarding security mechanisms, cryptographic primitives like ciphers, hash functions or Random Number Generators (RNGs) are pivotal. This article focuses on the design of an RNG that is a critical component in tasks such as the generation of a fresh session key or a set of random numbers for an authentication protocol. Although it is not within the scope of this article, it is worth mentioning that the sensors in WSNs due to their wireless communication capabilities are also vulnerable to disruptions of the radio channel. For instance, a DoS jamming attack can be easily executed by an adversary with a low-cost hardware, and ad hoc solutions, such as those presented in [11,12] or [13], are needed for its detection.

There are two main approaches to generate random numbers [14]. Firstly, computational algorithms, which depend on an initial value (seed or key), can be employed to generate long sequences of bits that look like a data stream generated by a random variable. These sorts of generators are called PseudoRandom Number Generators (PRNGs) [15]. Secondly, physical phenomena such as atmospheric noise or the thermal noise from a Zener diode can be used to generate random numbers due to their highly entropic nature [16]. Generators under this second approach are labeled as True Random Number Generators (TRNGs).

In this article, an innovative TRNG is proposed. As mentioned, a TRNG exploits a physical phenomenon. In our case, it takes advantage of an organ, particularly the heart, which is part of our bodies. The Electrocardiogram (ECG), which is the signal derived from the electrical activity of the heart and which can be measured through a sensor with several leads on our bodies, is the input to our system and from which true random bits are extracted. That means that each of us is the holder of a potential source of entropy just because our heart-beats to keep us alive. The details of the proposal are given in the following sections.

## 2. Motivation and Related Work

In the last few years, WSNs have attracted the attention of many researchers because of their great potential. These can be categorized depending on: (1) the place where the sensors are deployed (terrestrial, underground or underwater WSNs); (2) their ability to deal with multimedia data (multimedia WSNs); and (3) their ability to move around (mobile WSNs) [17]. The domains in which WSNs have been applied are very diverse. Monitoring and tracking are the two main purposes of the wide suite of applications [18]. Among the main fields of application are military, environment, industry, agriculture, urbanization, infrastructure and health. This work is framed within BANs, in which health (patient monitoring) is the star application [19]. In our particular case, the monitored vital signal is used for security purposes (random number generation); patient status monitoring can be done at the same time.

As mentioned, the security of sensors in WSNs is fundamental to the success of the IoT paradigm [20]. Cryptographic solutions must be supported on-board these devices, and random-number generators are one of the commonly-required cryptographic primitives. In this vein, the proposal takes advantage of the fact that some sensors record our vital signals. For this reason, it explores whether randomness can be extracted from physiological signals. In fact, some authors have recently studied this topic in the context of neuronal signals [21,22]. The main limitation of these studies is length of the recordings used and the fact that medical-purpose Electroencephalogram (EEG) sensors have limited portability capabilities.

**Figure 1:**
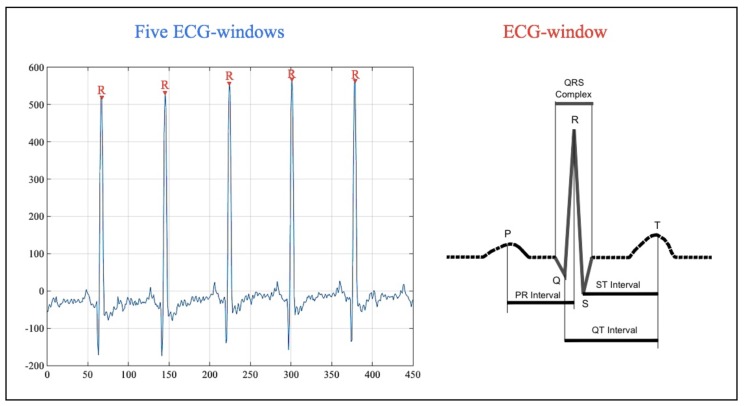
ECG signal.

In our case, the experiments focus on heart signals. Particularly, the electrical signal of the heart can be measured by placing electrodes (e.g., three or 12 leads) on the body of the subject under analysis. The representation of this signal is the Electrocardiogram (ECG). There are five characteristics points in the ECG: (1) the P-wave represents the depolarization of the atria; (2–4) the QRS complex represents the depolarization of the ventricles; and (5) the T-wave represents the re-polarization of the ventricles [23]. In Figure 1, an ECG signal and its characteristics points are sketched.

For cybersecurity purposes, the time interval between two consecutive heart-beats (R-peaks, which occur when the ventricles begin to contract), has gained the attention of many researchers in recent years [24,25,26]. This interval is commonly referred to as the Inter-Pulse-Interval (IPI). Accordingly, an interesting and proof-of-concept work can be found in [8], where Peter et al. presented a design and implementation of an IPI-based authentication protocol. In [27,28], the authors showed how IPI-based values can be employed as cryptographic keys. In addition, ECG biometrics is a growing field in which some approaches are based on characteristics’ points (including R-peaks and IPIs) [29,30].

In relation to random numbers, some authors have pointed out how the last four bits of IPI values are highly entropic [27,31]. Nevertheless, high entropy is a necessary, but not a sufficient condition to be considered a random variable. In Table 1, the results obtained in the analysis of a 10-MB file of IPI values (4 LSBbits per IPI) with the ENT suite [32] are shown. Although the entropy value is high, the chi-square test clearly shows that this file is not random. In line with this, in [33], the randomness quality of IPI values was scrutinized in-depth using 19 public datasets with healthy and unhealthy subjects. Two main conclusions were drawn from this study: (1) IPI values can generate short bit streams that behave as a random variable; and (2) large files with IPI values have poor randomness quality. In addition, the generation of random numbers based on IPI values offers very low performance, and although, this value is double in [34], the offered throughput is still low.

For all this, the designed ECG-based TRNG does not use the IPI approach and exploits all the wealthy entropic information contained in the entire ECG signal.

## 3. Materials and Methods

**Figure 2:**
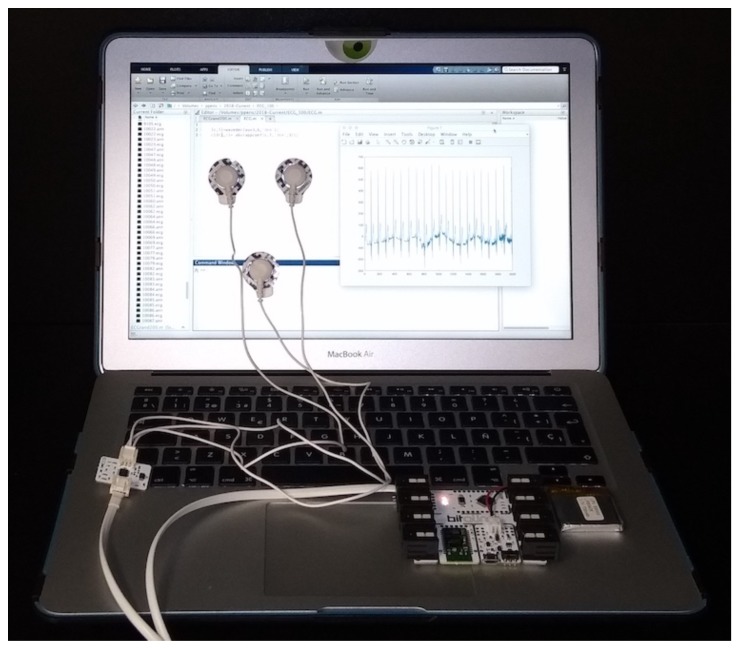
Hardware for building an ECG-based RNG.

In connection with the acquisition of the EEG signal, both medical equipment or low-cost sensors can be used for recording. The former ones often use twelve electrodes over the chest and limbs. These recordings are very accurate, but their portability is limited, making these devices unsuitable for WSNs. This equipment is commonly used in hospitals, and the subject must be at rest. With regard to low-cost sensors, only two or three electrodes on the chest or wrists are needed to capture the ECG traces. The signal can be a little noisy, but portability and integration into wearable devices (e.g., smart-watches or t-shirts [35]) make these devices very appropriate for WSNs: the wearer may be performing activities of her or his daily life; in other words, there is no need for the subject to be at rest. In our particular case, as a proof-of-concept, a low-cost ECG sensor (BITalino board with an ECG sensor [36]) was used for the acquisition of the ECG records. For this, three electrodes can be placed at the chest, but also at the palms of the hands. The aim of our contribution, taking advantage of the fact that some sensors in WSNs have the ability to sense heart signals, is to extract random numbers, which can be used for security purposes, from the above aforementioned signals. Once the raw ECG signal is acquired, pre-processing and randomness extraction by wavelet decomposition can be computed at the sensor itself or at the central node of the WSN that has greater computational and memory capabilities. Figure 2 shows all the necessary hardware, and the source-code is available in the following link to facilitate the reproduction of all the results (source code is available at these two links: https://goo.gl/WmQiiC and https://goo.gl/TpvSQq). The signal pre-processing and randomness extraction procedure are described below.

In detail, the ECG records have been cleaned using the following procedure (pre-processing procedure in Algorithm 1). Once the DC component is eliminated, a bandpass filter is used to remove two main noise sources. The lower and upper cut-off frequencies are fixed to 0.67 (to eliminate the noise caused by the respiration) and 45 Hz (to eliminate the power line noise), respectively.

**Algorithm 1** ECG-RNG.
1:**procedure**Preprocessing($ECG^{raw}$)2:    DC elimination3:    Bandpass filter ([0.67−45Hz])

4:**procedure**Wavelet decomposition($ECG^{cleaned}$)5:    Split $ECG^{cleaned}$ into ECG-windows (one heart-beat per window)6:    **for** each ECG-window(j) **do**7:        Discrete wavelet decomposition (set parameters *L* and wf)8:**procedure**Entropy extraction(Wavelet coefficients of each ECG-window (${\left\{c_{i}\right\}}_{i=1}^{N}$))9:    **for** each $c_{i}$ **do**10:        Fractional part extraction ($z_{i}$)11:        Output the 8-LBS bits (ri)


**Figure 3:**
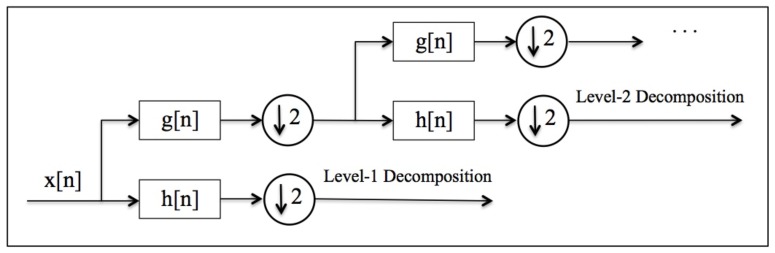
Wavelet decomposition of a signal x[n].

For squeezing random values from the clean ECG trace, the following procedure (wavelet decomposition procedure in Algorithm 1) is proposed. The ECG record is split into windows that contain an R-peak (one heart-beat); for each ECG record, the first and last fifty windows have been discarded to guarantee that the signal is properly registered. Secondly, the approximation coefficients of each EGG window are obtained by wavelet analysis. Note that the discrete wavelet transform of a signal (x[n]) is computed by passing it through a low-pass filter (g[n]) and and high-pass filter (h[n]). The signal is then sub-sampled by 2, and the process is repeated to increase the level of decomposition. In particular, the number of iterations is conditioned by the pursued decomposition level. The procedure is summarized in Figure 3; the reader can consult [37] for a detailed explanation.

As for the wavelet decomposition, there are two key-parameters that need to be set and a wide range of possibilities are studied in the following sections. On the one hand, *L* parameter sets the decomposition level: L={1,2,3,4} are the tested levels. On the other hand, wf sets the wavelet family (e.g., Daubechies or coiflets) used in the decomposition and determines the filters used (g[n] and h[n]).

Finally, random bits are extracted (entropy extraction procedure in Algorithm 1) using a kind of quantization. More precisely, the fractional part of each coefficient is converted into a 32-bit unsigned value, and then, the 8 LSB bits are extracted. Mathematically, let $c_{i}$ be a coefficient of wavelet-decomposition and $r_{i}$ an outputted random byte. Then,

(1) $$\begin{array}{ccc} z_{i} & = & uint32(\left(c_{i}\times 10^{4}\right)\gg 24)\\ r_{i} & = & z_{i}\left(0,\cdots ,7\right) \end{array}lll$$

Although the proposal was initially evaluated with ECG records obtained with the BITalino board, an in-depth analysis has been carried out, using a well-known and public dataset that uses three electrodes. More precisely, the E-HOL-03-0202-003 dataset, which was provided by the Telemetric and ECG Warehouse (THEW) of University of Rochester (dataset available at: http://thew-project.org/index.htm), has been employed. ECG records were acquired using three pseudo-orthogonal lead configuration (X, Y and Z), and the sampling frequency was set to 200 Hz. The descriptive statistics of the dataset are summarized in Table 2.

This database has features that are not present in many other public datasets. First, the number of individuals (ECG signals) is very large (i.e., 202 subjects; in our experiments, 3 ECG records were discarded due to the very short length of these recordings). Secondly, each ECG record lasts around 24 h, which is much longer than the length of ECG files available in many other public datasets. Finally, it is worth mentioning that the subjects were healthy, and therefore uniformity in the distribution can be assumed.

## 4. Results and Analysis

To assess the randomness quality of the outputted bits, three of the most common statistical tests batteries to evaluate the randomness quality of a RNG have been used: NIST [38], DIEHARDER [39] and ENT [32]. NIST is the most demanding battery and requires long files (several tens of megabytes). In our particular case, files with a size of around 100 MB have been generated. For each subject (199 in total), experiments lasted between 4 and 6 h (the time interval was randomly chosen from the 24 h available of the ECG signal), and therefore, 0.5-MB files were obtained per subject after the entropy extraction by wavelet analysis (see Section 3 for details). Finally, all the files were appended (assuming independent and identically distributed random variables), and this was the file analyzed; note that the NIST suite requires files of at least 30 MB that would require the recording of one individual during approximately 15 days. In relation to the parameters wf and *L*, Daubechies was the family used (the number of vanishing moments was set to N=4), and there were 1–4 levels tested.

Table 3, Table 4 and Table 5 summarize the results obtained with the NIST, DIEHARD and ENT suites for the four configurations studied. It is noteworthy that the NIST suite is devoted to test RNGs that have been designed for security purposes. Table 3 shows the *p*-value and the proportion of tests that pass each one of the fifteen tests included in the suite. Without a doubt, all configurations pass all the tests at the 0.005 level of significance, and it can be concluded that the output behaves as a random variable. Table 4 summarizes the *p*-values for each one of the test included in the Diehardsuite. The results were consistent with the NIST results. For a wavelet decomposition of three or four levels (the last two columns of Table 4), all tests passed. In the case of a decomposition of one or two levels, all the tests passed except a pair of tests where a weak-pass was obtained (*p*-value < 0.005); these are highlighted in bold in the table. Although the differences were minimal, the results indicated that a decomposition with a larger number of levels avoided the appearance of rare/weak patterns in the output. Finally, ENT results (as shown in Table 5) were in tune with all the above. In fact, and contrary to the results shown in Table 1 of Section 2, all tests were extensively passed. It is worth noting how the chi-square test was close to the optimal value (256).

In the subsequent subsections, the above analysis is rounded off by a bias and distinctiveness analysis. The performance of our proposal has also been analyzed and compared to previous works. Finally, some light is shed on which wavelet-family is more appropriate for the generation of random numbers.

### 4.1. Bias Analysis

**Figure 4:**
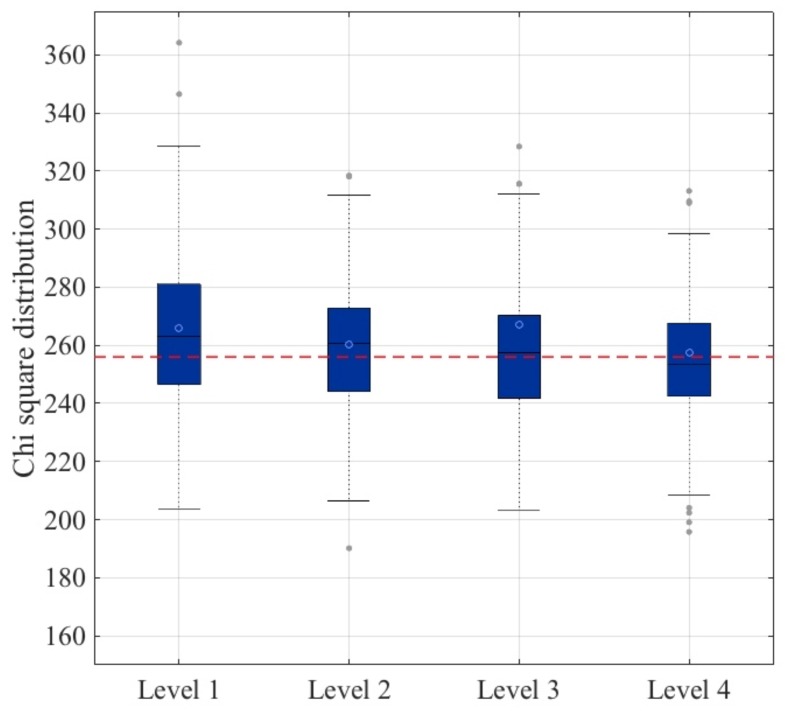
Bias analysis.

The bias of the outputted stream has been analyzed for each approach. To this end, the following experiment has been carried out. For each subject (199 in total), a file of 0.5 MB has been generated using the same procedure as described in Section 3 and analyzed using the ENT suite. In Figure 4, a box-plot of the chi-square values is shown. It is worth noting that the optimal value of the chi-square test was 256, and the greater the distance to this optimal value, the greater the bias in the data. Using this analysis, it could be concluded that the fourth level approach was the most appropriate to build a secure and robust TRNG based on ECG signals: the average value (blue circle) was the optimal one, and the distribution of values between the first and third quartile was the narrowest.

### 4.2. Distinctiveness Analysis

**Figure 5:**
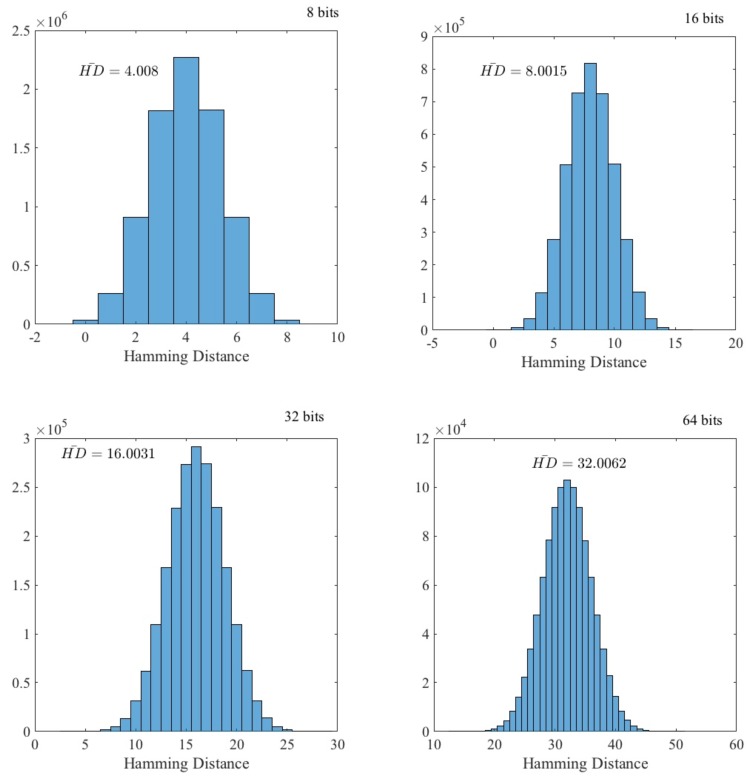
Hamming distance distribution.

We have tested whether the random data generated from different ECG signals (each one belonging to a single individual) were distinct. If this holds, an adversary cannot use data from another individual to predict values generated by the target. To evaluate this, as in Section 4.1, a file of 0.5 MB has been generated for each individual (ECG record). Then, for each file, data were grouped into {8,16,32,64}-bit words. In Figure 5, the data distribution of the Hamming distance between all the pairs ($C_{199,2}$ in our particular case) of individuals belonging to the dataset is shown. As expected, the distribution fit a binomial distribution:

(2) p(k)=nkpk(1−p)n−k

where n={8,16,32,64} and p=1/2. The experimental mean value of *k* is {4.0008,8.0015,16.0031,32.0062}, which is nearly the expected value (E(k)=np={8,16,32,64}×(1/2)={4,8,16,32}). From all this, it can be concluded that the advantage for an adversary to predict values using ECG signals from a different individual was zero.

Apart from using the ECG of a different user, the attacker may be tempted to capture ECG signals from a distance. In [40], Calleja et al. showed how IPI-values (R-peaks) can be eavesdropped without touching the target individual and using a camera. Fortunately, this approach is totally uselessness against our proposal since the whole ECG signal (P-wave, QRS-complex and T-wave) was used and there is no way to predict or capture an entire ECG signal from a distance.

### 4.3. Performance Analysis

Apart from the poor randomness quality of IPI-based approaches [33], the throughput is also a bottleneck. Generally, in this sort of approach, four random bits (LSB) are extracted after the observation of two heart-beats [27,31]. In order to improve efficiency, in a recent proposal, Pirbhulal et al. were able to extract 16 bits per IPI value [34]. Despite all efforts, IPI-based approaches suffer from low throughput. Luckily, our approach was much more efficient since it was possible to extract several random bytes per each heart-beat. In Table 6, the performance of existing approaches is summarized. To facilitate the understanding of these values, in Columns 3 and 4, a healthy individual whose heart-beats between 60- and 100-times per minute (i.e., [1–0.6] s per beat) is assumed.

**Figure 6:**
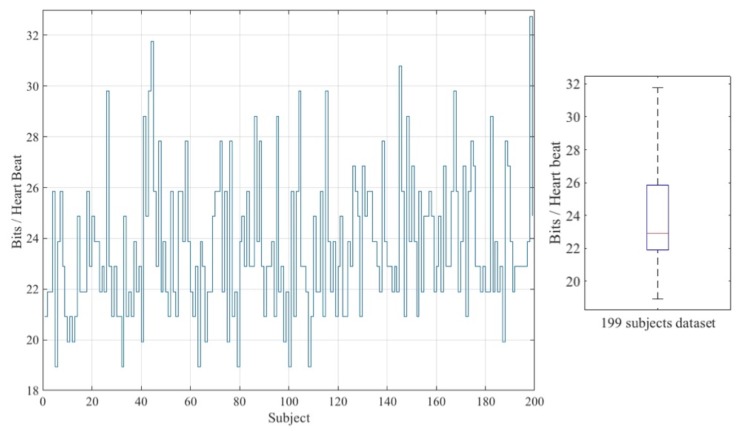
Throughput analysis.

The particular number of bits that can be extracted from an ECG-window (including only one R-peak) depends on the heart rate of the individual. Figure 6 shows the average value of bits extracted per heart-beat for each of the 199 subjects belonging to the dataset. The overall average value of all these values is the value shown in Table 6.

Compared to previous proposals, the advantages of our solution were two-fold. On the one hand, one heart-beat (ECG-window) is only needed to extract random bits; note that IPI approaches require two heart-beats since a difference between two R-peaks is computed. On the other hand, the throughput has increased drastically (with a 2200% increase in the worst-case scenario). Therefore, our proposal was able to generate random bits at a moderately high throughput rate.

### 4.4. Wavelet Family Analysis

The wavelet decomposition of the ECG signal represents the core function of the proposed RNG. Up to this point, the experimentation has been conducted using the Daubechies family (the number of vanishing moments is set to four; N=4). For completeness, we have evaluated the RNG with other families (i.e., Haar, coiflets, symlets, discrete Meyer, biorthogonal) to discern which alternatives were the most appropriate for the generation of randomness. A file of 100 MB has been generated in each case and then evaluated using the ENT, DIEHARDER and NIST randomness test suites. Table 7 summarizes the overall average results.

**Figure 7:**
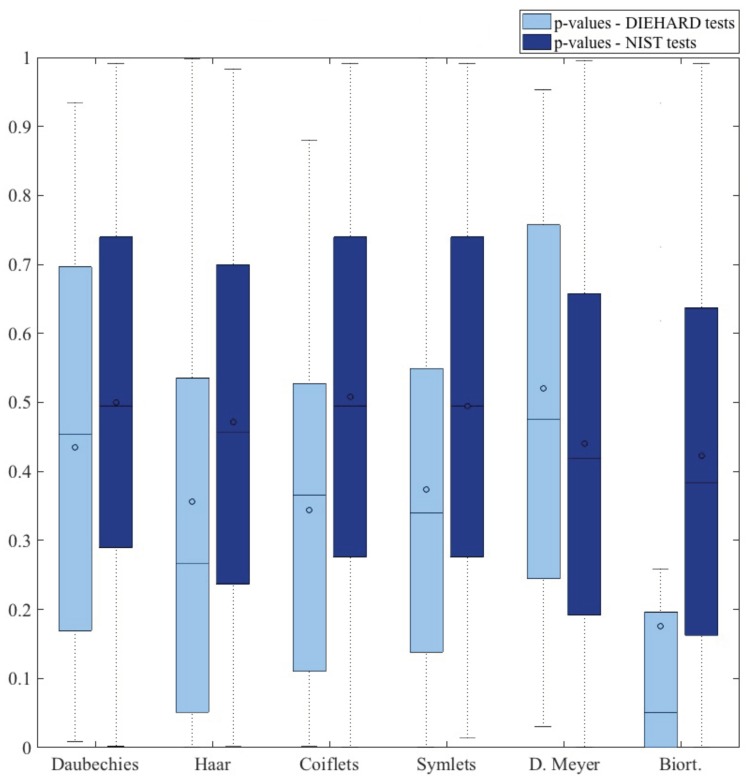
*p*-values (DIEHARD and NIST suite tests).

As already mentioned, Daubechies was our first approach since this is the common mother wavelet used for the analysis of the ECG signal [41,42,43]. Nevertheless, this paper explores how to extract randomness from ECG signals by multi-level wavelet decomposition, and to the best of our knowledge, this is the first time this approach has been studied. Therefore, the choice of the most appropriate wavelet family has not been evaluated before in the context of random number generation. Figure 7 summarizes the distribution of *p*-values for the tests included in the DIEHARD and NIST suites in order to gain a better overall perspective of the results. The biorthogonal approach can certainly be ruled out as the *p*-values were far away from an uniform distribution. In addition, Haar, coiflets and symlets are also not recommended as the median of the *p*-values fell well bellow the optimal value of 0.5. For all this, the use of Daubechies or discrete Meyer is recommended since with both approaches, the output stream behaved as a random variable.

## 5. Discussion

Regarding the extraction of randomness from cardiac signals, the reader may be tempted to think that this topic has already been studied in the literature. Nevertheless, there is a key-difference between IPI-based approaches, such as [31,34], and our proposal. In the former, the time difference between two R-peaks is the only information used; note that R-peaks can be read from an ECG record, but also from a Photoplethysmography (PPG) signal. In our approach, a whole ECG trace (P-wave, QRS-complex, T-wave) is needed.

As mentioned, the entire ECG signal is used to extract randomness from an ECG-window. In particular, a multi-level decomposition by wavelet analysis is the chosen technique. To the best of our knowledge, this is the first time that this approach has been proposed. It is worth noting that other transform domains (e.g., Fourier or Hadamard) have been tested, but the results were not as good as in the wavelet domain. Regarding the mother wavelet, as shown in Section 4.4, the two recommended families are Daubechies or Meyer.

The experimentation has been conducted with the E-HOL-03-0202-003 dataset, which contains 202 subjects recorded over a 24-h period. In the above-mentioned dataset, the subjects were healthy. The proposal could have been tested with a dataset in which the subjects suffer from a cardiac ailment. Nevertheless, this would be a more advantageous scenario since the disease itself would introduce more entropy into the ECG signal. Therefore, in a healthy setting, the worst case scenario for random number generation is considered.

Another critical aspect of the proposal is whether an adversary could predict the values of a target user using another user’s ECG. The experiments conducted in Section 4.2 clearly point out how the attacker has no chance of success; that is, the adversary’s advantage is zero. Furthermore, and unlike IPI-based approaches, in the proposed TRNG, the usage of the entire ECG signal prevents attacks where the heart signal is eavesdropped from a distance.

Finally, apart from randomness, throughput is a key aspect for cryptographic primitives. The proposed TRNG far exceeds its predecessors: throughput rate (bytes/s) is multiplied by about 20 in the worst-case scenario. Despite this increment, the study of whether the ECG signal can be further squeezed to extract randomness is a pending work.

## 6. Conclusions

In the last few years, the e-health sector has undergone a major transformation. The population is more concerned about it habits and health and has access to detailed information thanks to the wide variety of low-cost sensors or medical devices that monitor our vital signals and daily activities; all these sensors together with a central gateway make up the WSN and, more particularly, the WBAN when the sensors surround our bodies. In addition, the new generation of medical devices (e.g., pacemakers or insulin pumps) monitor physiological signals and upload these data to the hospital cloud. The doctor can not only check the status of a patient in real time, but can also re-program the device while the patient is comfortably at home. In short, the new health-tracking or IoT medical devices aim to improve the quality life of citizens by improving our performance/habits or treating a disease.

The benefits associated with continuous monitoring of our vital signals for medical or performance purposes are well-known. Nevertheless, the situation is very risky if security is not included on-board (and preferably by design) in these sensors/devices within a WSN. Therefore, sensors in a BAN that monitor our vital signals can be used with a dual purpose. On the one hand, the main goal is to improve the health of the user. Besides, additional goals do not have to compromise this primary goal; note that some sensors are critical for the treatment of certain medical conditions (e.g., heart attacks or epileptic seizures). On the other hand, the wireless connectivity of the devices makes the incorporation of security protection mechanisms mandatory; RNGs, such as the one designed in this article, can help in this task.

An authentication protocol is one of the most common solutions to provide an adequate security level for sensors with limited capabilities (computation, storage and energy). For this purpose, RNGs may be necessary for the generation of random numbers included in a cryptographic protocol or for the seed(s) employed in a key generation algorithm. As mentioned, in the context of random number generation, TRNGs exploit a physical phenomenon from which they extract entropy. Based on this principle, this article explores whether the randomness from cardiac signals can be extracted. In detail, a wavelet decomposition has been used to extract randomness from an ECG-window. To the best of our knowledge, this is the first time that this approach has been proposed. From the analysis carried out, it is concluded that the output of the proposed ECG-based TRGN behaves as a random variable. In addition, our TRNG offers a high throughput that has nothing to do with the low throughput of IPI-based approaches.

As future work, the proposal can be tested with other vital signals such as respiration, blood pressure or even an electroencephalogram. There is also room to study in depth the entropy extraction problem in a transformed domain.

## Figures and Tables

**Table 1:** ENT results (10-MB file with 4-LSB Inter-Pulse-Interval (IPI) values).

Approximately	IPI-Based Approach	Optimal Values
Entropy	7.957724	8
Optimum	0%	0%
compression		
Chi square	493.49	256
	(0.01%)	([5–95%])
Arithmetic mean value	123.0993	127.5
Monte Carlo π value	3.158811	3.14159
Serial correlation	0.031878	0
coefficient		

**Table 2:** Population descriptive statistics.

Statistic	Male	Female
Number	101	101
Height	176.8	162.3
Weight	77.6	62.3
Body Mass	24.7	23.7

**Table 3:** NIST results.

Approximately	Level 1	Level 2	Level 3	Level 4
Frequency	0.8165 (49/50)	0.9558 (50/50)	0.0200 (49/50)	0.8514 (49/50)
Block Frequency	0.4190 (49/50)	0.4190 (47/50)	0.8832 (49/50)	0.1917 (49/50)
Cumulative Sums	0.5207 (2/2)	0.4356 (2/2)	0.6101 (2/2)	0.1563 (2/2)
	(49/50)	(50/50)	(49/50)	(49/50)
Runs	0.6993 (48/50)	0.6993 (50/50)	0.4944 (50/50)	0.4559 (50/50)
Longest Run	0.2897 (50/50)	0.6993 (50/50)	0.9915 (50/50)	0.8832 (50/50)
Rank	0.08559 (50/50)	0.5341 50/50	0.3505 (49/50)	0.0352 (50/50)
FFT	0.1223 (50/50)	0.0757 (49/50)	0.5749 (49/50)	0.2897 (50/50)
Non-Overlapping	0.4986 (148/148)	0.4881 (148/148)	0.5080 (148/148)	0.5090 (148/148)
Template	(>49/50)	(>49/50)	(>49/50)	(>49/50)
Overlapping Template	0.3838 (50/50)	0.1719 (48/50)	0.9558 (48/50)	0.4190 (49/50)
Universal	0.3505 (50/50)	0.0156 (50/50)	0.3838 (48/50)	0.9915 (49/50)
Approximate Entropy	0.0669 (48/50)	0.9558 (49/50)	0.6993 (50/50)	0.1088 (50/50)
Random Excursions	0.2865 (8/8)	0.1094 (8/8)	0.3629 (8/8)	0.4111 (8/8)
	(>36/38)	(>37/38)	(>33/34)	(>32/33)
Random Excursions Variant	0.2867 (18/18)	0.3328 (18/18)	0.4612 (18/18)	0.3969 (18/18)
	(>36/37)	(>37/38)	(>33/34)	(>32/33)
Serial	0.6511 (2/2)	0.9537 (2/2)	0.1753 (2/2)	0.5116 (2/2)
	(>49/50)	(50/50)	(49/50)	(49/50)
Linear Complexity	0.0352 (50/50)	0.2622 (50/50)	0.5749 (49/50)	0.9717 (50/50)

**Table 4:** Diehardresults.

Approximately	Level 1	Level 2	Level 3	Level 4
Birthdays	0.68301545	0.61270139	0.80007480	0.94460956
OPERM5	0.01657098	0.76376607	0.77095792	**0.0012866**
32 × 32 Binary Rank	0.73054931	0.93907677	0.93485678	0.40762130
6 × 8 Binary Rank	0.03964233	0.63609809	0.01640541	0.78004161
Bitstream	0.44644237	0.38432822	0.76304154	0.46452841
OQSO	0.16901300	**0.0000523**	0.10390905	0.07871345
	0.76574765	0.63218487	0.56716581	0.69843874
DNA	0.01104271	0.66337412	0.04864965	0.16432922
Count the 1’s (stream)	0.64310466	0.75768749	0.14166650	0.64535121
Count the 1’s Test (bytes)	0.61217963	0.12233837	0.45342646	0.31039533
Parking Lot	0.01700299	0.72327165	0.45123033	0.61550204
Minimum Distance	0.05835137	0.39712445	0.57168207	0.60978869
(2D Circle)				
3D Sphere	0.45525876	0.40382693	0.74404666	0.94736187
(Minimum Distance)				
Squeeze Test	0.51553404	**0.0000231**	0.26298106	0.87828628
Runs	0.01450632	0.17897685	0.64894698	0.85809732
	0.77031157	0.78097772	0.51236956	0.27052895
Craps	0.01027903	0.09666884	0.00901385	0.91551334
	**0.0042827**	0.08596808	0.27730790	0.90795457

**Table 5:** ENT results.

Approximately	Level 1	Level 2	Level 3	Level 4
Entropy	7.999998	7.999998	7.999998	7.999998
Optimum	0 %	0 %	0 %	0 %
Compression				
Chi Square	279.22	268.41	235.82	313.44
	(14.24 %)	(26.99 %)	(80.01 %)	(0.73 %)
Arithmetic Mean Value	127.4657	127.4731	127.4896	127.4931
Monte Carlo π Value	3.141955902	3.142772504	3.141912708	3.141860883
Serial Correlation	−0.000105	0.000022	−0.000124	0.000058
Coefficient				

**Table 6:** Performance analysis.

Approach	Efficiency	Throughput (60 PPMs)	Throughput (100 PPMs)
IPI-based approaches [27,31]	4 bits/2 heart-beats	2 bits /second	3.3 bits/second
Pirbhulal et al. [34]	16 bits/2 heart-beats	8 bits/second	13.33 bits/second
Our approach	23 bytes/heart-beat	184 bits/second	306 bits/second

**Table 7:** Wavelet family analysis.

	Test	ENT	DIEHARDER	NIST
Family				
Daubechies (*N* = 4)		PASS (6/6)	PASS (15/15)	PASS (15/15)
Haar		PASS (6/6)	(12 PASS–2 WEAK–1 FAILED)/15	PASS (15/15)
Coiflets (*N* = 3)		PASS (6/6)	(14 PASS–1 WEAK) /15	PASS (15/15)
Symlets (*N* = 4)		PASS (6/6)	(13 PASS–2 WEAK) /15	PASS (15/15)
Discrete Meyer		PASS (6/6)	PASS (15/15)	PASS (14/15)
Biorthogonal ($N_{r(d)}=3$)		PASS (6/6)	(13 PASS–2 WEAK) /15	PASS (12/15)
